# Editorial: Pride in Frontiers in Human Neuroscience

**DOI:** 10.3389/fnhum.2025.1754283

**Published:** 2026-01-14

**Authors:** Riccardo Manca, Lucas Albrechet-Souza, Jhon Alexander Moreno

**Affiliations:** 1 Department of Medicine and Surgery, University of Parma, Parma, Italy; 2 Department of Cell Biology and Anatomy, Louisiana State University Health Sciences Center, New Orleans, LA, United States; 3 Department of Psychology, Université de Montréal, Montreal, QC, Canada; 4 Centre de Recherche de l'Institut Universitaire de Gériatrie de Montréal, Innovation, Technology, and Cognition Research Laboratory (INTECOG), Montreal, QC, Canada

**Keywords:** LGBTQIA+, gender identity, sexual orientation, transgender health, neuroscience, brain health, health disparities, gender-affirming care

Pride is a multifaceted emotion grounded in honor, dignity, and self-respect, arising not only from personal accomplishments but also from the achievements of those with whom we share identity and community. This emotion is often amplified when success follows adversity, a dynamic that resonates deeply with the histories of many minority groups, particularly lesbian, gay, bisexual, transgender, queer, intersex, asexual, and people with other sexual orientations and forms of gender expression (LGBTQIA+). Research in the broad field of neuroscience has contributed to highlight issues central to LGBTQIA+ experiences, including those faced by LGBTQIA+ scientists (Freeman, 2021), and has begun to outline evidence-based strategies to address them. The *Pride in Frontiers in Human Neuroscience* Research Topic brings together a historical overview of neuroscience research involving LGBTQIA+ populations and highlights recent scientific advances with clinical relevance for the mental, cognitive, and neurological health of this community, with particular attention to transgender and gender diverse individuals.

Moreno et al. provide a comprehensive overview of the historical trajectory of neuroscientific research on sexual and gender diversity. Their analysis shows that this line of inquiry began in the 1910s, with early publications largely characterized by pathologizing interpretations of non-heterosexual behavior. This negative bias toward homosexuality persisted for several decades. It was only in the 1970s that the field began a gradual shift away from searching for neural “correlates” of sexuality—and, to a lesser extent, gender identity—toward research questions more directly relevant to the clinical needs and lived experiences of LGBTQIA+ people. These developments ultimately paved the way for more recent investigations examining how the social challenges faced by LGBTQIA+ individuals may adversely affect a broad range of health-related outcomes.

A novel perspective on the experimental study of gender identity and gender roles emerges from the use of metaverse-based approaches (Kang and Rhee). In their study, the authors examined how adopting male avatars affected female participants' experiences and self-perceptions. Interacting through a male avatar not only enhanced participants' momentary identification with male gender roles but also deepened their understanding of gender dynamics and increased empathy toward gendered experiences. These results highlight the metaverse as an innovative methodological tool with potential applications in interventions designed to reduce gender-related biases and foster greater awareness of gender diversity.

Kaprinis and Charalampakis review and synthesize the most influential theoretical frameworks describing how social exclusion affects LGBTQIA+ people (i.e., the Rejection Sensitivity Model, the Minority Stress Model, and the Psychological Mediation Framework). These models consistently show that chronic stress and rejection linked to minority status exert pervasive effects on health, particularly mental health, and can even give rise to detectable neurobiological changes. The authors discuss important limitations of existing models and call for expanding the field by integrating themes such as intersectionality (i.e., race, class, ability), post-intervention neurological healing, and multilevel analytical approaches. They highlight the need to prioritize community-based participatory research to illustrate the needs and voices of LGBTQIA+ individuals throughout the research process.

Intersectionality among sexual orientation, gender identity, and neurodiversity was further explored by Kroll et al., with a specific focus on mental health. In a large sample of individuals enrolled in a virtual intensive mental health treatment program, participants who identified as neither female nor male and those identifying as pansexual were the most likely to report being neurodivergent. The study also found that mental health outcomes were poorer among participants who did not identify within the binary genders and among non-heterosexual individuals, regardless of whether they were neurotypical or neurodivergent. These findings underscore the need for mental health interventions that are tailored to the unique experiences and challenges of LGBTQIA+ communities.

Tornese et al. review the evidence on the mental health effects of gonadotropin-releasing hormone agonist treatments in transgender and gender diverse youth. Their synthesis indicates that these treatments are associated with significant improvements in mental health, particularly when this therapy is followed by gender-affirming hormone interventions. Importantly, the authors emphasize that counseling and psychosocial support remain essential components of clinical care. They also highlight the need for ongoing monitoring of bone health, neurocognitive development, and potential cancer-related risks as part of a comprehensive management plan.

Three articles in this Research Topic focus specifically on brain health within the LGBTQIA+ community. Manca et al. synthesize current evidence on disparities in cognitive decline and dementia risk, emphasizing the considerable limitations and gaps in the existing literature. Notably, they identify a striking scarcity of neuroscientific research involving older LGBTQIA+ adults and offer recommendations for advancing this emerging field, including developing and testing specific hypotheses, refining measures of risk and protective factors, and improving cognitive outcome assessments across diverse LGBTQIA+ groups. The remaining two articles concentrate on the transgender population. Wright and Murphy review the literature on the relationship between gender-affirming hormone replacement therapy and cerebrovascular health in transgender people. While both human and preclinical studies have reported potential effects of hormone therapy on cerebrovascular outcomes, the evidence for increased cerebrovascular risk in transgender women remains inconsistent across studies. Insaurralde et al. contribute to this discussion by presenting the case of a 30-year-old transgender woman who experienced a stroke, possibly because of unsupervised estrogen use. Since this transgender woman had no known stroke risk factors, the authors concluded that self-administered hormone therapy was likely a primary contributing factor. This case highlights a key consideration in the broader debate: although contemporary gender-affirming hormone regimens are generally safe, medical oversight is essential to minimize preventable risks.

Overall, the *Pride in Frontiers in Human Neuroscience* Research Topic provides a timely snapshot of emerging neuroscientific findings relevant to diverse LGBTQIA+ communities, highlighting a growing recognition of mental, cognitive, and brain health disparities. With studies from the perspectives of neuroscience, psychology, medicine, and social sciences, the Research Topic contributes uniquely to advancing and deepening the scientific understanding of LGBTQIA+ neuroscience. The content features research from three continents, adopting a lifespan perspective addressing the health of LGBTQIA+ youth, mid-age, and older adults. The studies featured here complement prior evidence that some LGBTQIA+ populations may face elevated risks for certain neurological conditions, such as multiple sclerosis (Pakpoor et al., 2016) and epilepsy (Johnson et al., 2024), with concerns regarding cerebrovascular health of transgender individuals undergoing gender-affirming hormone therapy. Although neuroimaging investigations of brain health in LGBTQIA+ people remain limited (Manca et al., 2022; Manca and Venneri, 2020), this field is still in its infancy and is expanding rapidly. Together, these insights underscore the urgent need for continued, rigorous investigation into the biological and environmental factors that shape brain health in LGBTQIA+ people (Moreno et al., 2024). In parallel, innovative technological applications could provide promising tools to inform educational programs centered on gaining awareness of sexual and gender diversity and to develop evidence-based interventions promoting the wellbeing and flourishing of LGBTQIA+ individuals worldwide.
